# Arthroscopic All‐Inside Meniscal Tying for Anterior Horn Bucket‐Handle Meniscal Injuries

**DOI:** 10.1002/atn2.70267

**Published:** 2026-10-05

**Authors:** Ziyad El Qirem, Wael Al‐Atout, Mohamad Makahleh, Barakat Elsaqa

**Affiliations:** ^1^ Department of Orthopedic Surgery The Specialty Hospital Amman Jordan

## Abstract

Anterior horn meniscal tear is a relatively uncommon injury to be seen in our knee arthroscopy surgeries. It can be a part of a bucket‐handle tear or less commonly in isolation. Several repair techniques have been used to treat this injury, with the most commonly used ones being the outside‐in and inside‐out techniques, with the disadvantage of opening a 1‐ to 2‐cm skin incision and a possible knot irritation. We describe an all‐inside arthroscopic technique for an anterior horn bucket‐handle medial meniscus tear using an arthroscopic retrieval and a suture passer to perfectly pass the repair wire and then tying it arthroscopically. We believe that this technique has the advantage of avoiding any skin complications. Also, it allows more control over the passing location of the repair wire to allow good reduction and repair of the meniscal tear.

## Graphical abstract

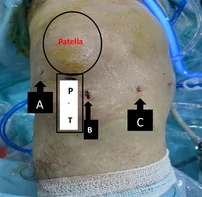

**VIDEO 1:** Arthroscopic all‐inside meniscal repair for anterior horn bucket‐handle meniscal injuries using a SutureLasso device. This video is a case example of an arthroscopic right knee bucket‐handle meniscus repair using 3 portals: high anterolateral portal, anteromedial portal, and far medial portal. The following steps are included: (1) arthroscopic evaluation, (2) trimming of the torn meniscus edges, (3) application of traction stitch, (4) reduction of the meniscus, (5) repairing of the posterior horn using all‐inside devices, (6) repairing of the meniscal body and anterior horn using an all‐inside technique with a help of a SutureLasso device, and (7) final retensioning of all sutures before cutting. Video content can be viewed at https://doi.org/10.1002/atn2.70267.

Meniscal injury is a common intra‐articular knee injury, especially among athletes.^1^ Thus, management of such injuries has evolved significantly over the past several decades. Menisci are fibrocartilaginous structures that were previously thought to be vestigial remnants of no function.^1^ The menisci are nowadays regarded as important intraarticular knee structures that play a role in stability, force transmission, and load distribution.^2^, ^3^, ^4^ This led to the introduction of meniscal repair, which would restore the meniscus function in a better way.

Out of the meniscal injuries, anterior horn meniscal injuries are most commonly repaired using outside‐in and inside‐out techniques, which can carry a risk of knot irritation or prominence. Here, an all‐inside repair would be a better option for avoiding these complications.

In this technical note, we describe the technique of all‐inside repair of a bucket‐handle medial meniscus tear with extension to the anterior horn. The anterior horn all‐inside repair was achieved using the SutureLasso (Arthrex, Naples, FL) suture passer.

## SURGICAL TECHNIQUE

### Preoperative Evaluation

Patients with meniscal tears will present with a relatively swollen and painful knee with possible mechanical symptoms (locking and clicking), especially with squatting. Joint line tenderness and McMurray's test are usually positive. Diagnosis can be made with the help of a magnetic resonance imaging, as it delineates the injury in a better way and evaluates any associated injuries, such as anterior cruciate ligament injury.

### Setup

The patient is placed supine on the operative table under general anesthesia, and an upper thigh tourniquet is used. Knee is kept on 90° flexion on the table with a fixed roller under the foot distally. Then, after painting and draping the knee, we mark our arthroscopic portals in the operative knee.

### Portals

This technique was done using a 30° arthroscope on an anterior horn medial meniscus tear. Three arthroscopy portals to the knee were done: high anterolateral portal, mid‐portal just (medial to the patellar tendon), and far medial portal (working portal) (Figure 1). The operative technique is shown in Video 1.

**FIGURE 1:**
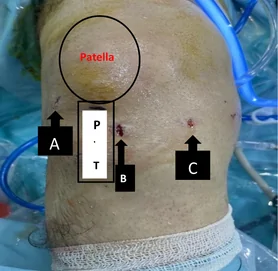
The right knee is shown. Three portals were marked: (A) high anterolateral portal, (B) mid‐portal, and (C) far medial portal. (P.T, patella tendon.)

### Operation

#### Diagnostic Arthroscopy

A normal diagnostic knee arthroscopy to the knee is performed. Starting from the high anterolateral portal, we established the mid‐portal just medial to the patellar tendon for shaving the Hoffa fat pad and probing the torn meniscus to assess its reduction and how to repair it (Figure 2). Medial collateral ligament pie crusting was done using an 18‐guage spinal needle, from its femoral insertion toward the posterior condyle, to enhance visualization.

**FIGURE 2:**
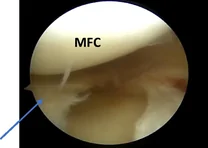
The right knee is shown. Viewing from the anterolateral. A medial meniscus bucket‐handle tear is shown (arrow). (MFC, medial femoral condyle.)

#### Meniscus Debridement and Reduction

After the diagnostic arthroscopy, an arthroscopic shaver was used to debride the edges of the torn meniscus, and then, a meniscal rasp was used to refresh the edges for better healing. After that, a far medial portal (a bit higher from the meniscus) was established. A stay suture is placed in the body of the torn meniscus using a Scorpion device (Arthrex) via the far medial portal to achieve temporary reduction before we place the first all‐inside suture. After the reduction of the torn meniscus, repair of the bucket‐handle meniscal tear is carried out from the posterior horn of the meniscus using all‐inside suture devices such as Air+ Meniscal Repair System (Stryker, Kalamazoo, MI) (Figure 3a,b). Then, the viewing portal is switched to the mid‐portal to repair the body and the anterior horn of the meniscus using an all‐inside technique.

**FIGURE 3:**
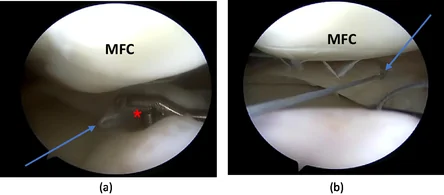
The right knee is shown. Viewing from the mid‐portal. (a) A stay suture (arrow) was taken (with the help of a scorpion device (asterisk)) in the body of the torn meniscus to help in meniscus reduction. (b) Repair of the posterior part of the meniscus with all‐inside devices (arrow). (MFC, medial femoral condyle.)

#### Anterior Horn Meniscal Repair

After switching the viewing portal to the mid‐portal, retrieving of the all‐inside suture wires is done from the anterolateral portal to allow final tensioning at the end. Then, a 45° SutureLasso (Arthrex) device is introduced from the far medial portal, and arthroscopic retrieval is introduced from the high anterolateral portal. The SutureLasso (Arthrex) device is passed sequentially from right‐to‐left direction in the torn meniscus with the help of an arthroscopic retrieval, first through the peripheral attachment part of the torn meniscus and then again through the displaced torn portion of the anterior horn of the medial meniscus. Feeding of the SutureLasso (Arthrex) loop is done and retrieved with the help of an arthroscopic retrieval (Arthrex) from the anterolateral portal (Figures 4a,b and 5). Shuttling of a size zero fiber wire (Arthrex) was performed through the SutureLasso (Arthrex) loop, and then, SutureLasso (Arthrex) device was removed.

**FIGURE 4:**
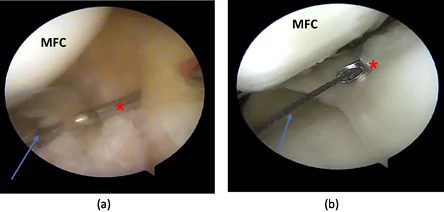
The right knee is shown. Viewing from the mid‐portal. (a) SutureLasso device (asterisk) was introduced through the far medial portal and passed sequentially in the torn meniscus with the help of suture retrieval (arrow). (b) Feeding of the SutureLasso device wire (arrow) to pass the repair fiber wire through it. (MFC, medial femoral condyle.)

**FIGURE 5:**
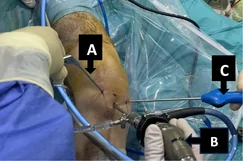
The right knee is shown, illustrating how it looks from the outside. (A) Arthroscopic retrieval passed from the anterolateral portal, (B) the mid‐portal is the viewing portal, and (C) the SutureLasso device passed from the far medial portal.

#### Arthroscopic Tying

The 2 ends of shuttled fiber wire were retrieved through the far medial portal, making sure that there was no soft‐tissue interposition (Figure 6). An arthroscopic knot pusher is used to tie the repair wires using the half‐hitch knot (Figure 7a,b). Before cutting any wires, sequential tightening of all sutures (including the all‐inside sutures) is performed with a final pull, after which they are cut. Repair is examined again using a probe from the far medial portal to confirm stability. A microfracture is performed using an awl with a 45° tip (Arthrex) near the lateral wall of the intercondylar notch, anterior to the femoral attachment of the anterior cruciate ligament to enhance healing.

**FIGURE 6:**
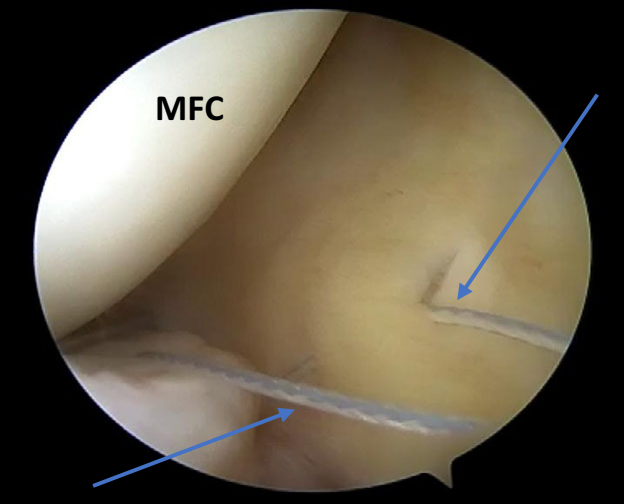
The right knee is shown. Viewing from the mid‐portal. Retrieval of the 2 ends of the shuttled fiber wire (arrow) through the far medial portal and making sure that there is no soft‐tissue interposition. (MFC, medial femoral condyle.)

**FIGURE 7:**
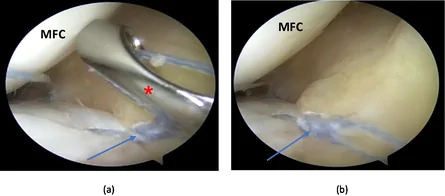
(a, b) The right knee is shown. Viewing from the mid‐portal. Tying of 2 ends of the fiber wire with a half‐hitch knot (arrow) with the help of a knot pusher (asterisk). (MFC, medial femoral condyle.)

## DISCUSSION

Considering history, partial or complete meniscectomy was the most popular procedure performed to treat knee pain caused by meniscal injury.^5^ However, with advances in research and arthroscopy techniques, follow‐up for patients who underwent such procedures revealed detrimental effects on cartilage and the quick development of osteoarthritis.^5^ This led to the introduction of meniscal repair, which is becoming increasingly indicated as it proved to be protective of the cartilage and exhibits lower rates of progression to osteoarthritis and better patient‐reported outcomes.^3^, ^4^, ^6^, ^7^ Arthroscopic meniscus repair shows high success rates and acceptable failure and reoperation rates, supporting its continued use in clinical practice.^4^

Advances in arthroscopic repair techniques for different patterns of meniscal injury are constantly arising, including all‐inside, outside‐in, and inside‐out techniques, each of which can be used in different suture configurations.^2^, ^3^ All‐inside repair is increasingly being used for meniscal repair.^8^ All‐inside repair devices and techniques are less technically demanding, decrease operative time, and provide comparable results to the gold standard inside‐out technique, with limitations in anterior horn meniscal injuries.^3^, ^8^ Despite having the risk of implant failure and iatrogenic cartilage injury, all‐inside repair has proven to provide earlier postop recovery and decreased risk of nerve injury compared with inside‐out repair.^3^ Although all‐suture and all‐inside repairs have advantages over their counterparts, they are costly, limiting their widespread use,^9^ particularly in resource‐constrained settings.

To overcome this, here, we describe a cost‐effective all‐inside arthroscopic technique for anterior horn medial meniscus tear using a SutureLasso (Arthrex) device and an arthroscopic retrieval (Arthrex).

Pearls and pitfalls of this technique are listed in Table 1.

**TABLE 1:** Pearls and Pitfalls

**Step**	**Pearls**	**Pitfalls**
1) Opening a far medial portal	A) Provides easy access to pass the SutureLasso device	A) Placing the 2 medial portals close to each other can cause difficulty in instrument handling and suture management B) Make sure to place this portal a bit higher from the meniscus to allow easy access of the SutureLasso device
2) Sequential passage of the SutureLasso device through the 2 ends of the torn meniscus	A) Allow more control on the SutureLasso device B) Allow more anatomical reduction of the torn meniscus	A) Needs special instruments to pass the SutureLasso like an arthroscopic retrieval
3) All‐inside arthroscopic repair and final tensioning of fiber wires	A) Avoids knot issues in the skin, for example, prominence B) Does not require a cannula usage C) Retensioning provides more secure fixation	A) Needs skills in arthroscopic knot tying and a special instrument like a knot pusher B) Remember to cut all repair wires last and to shuttle them through the anterolateral portal to allow better visualization during your repair

A few limitations of this technique include a possible learning curve for the surgeon to handle the suture passer. The advantages and disadvantages of this technique are listed in Table 2.

**TABLE 2:** Advantages and Disadvantages

**Advantages**	**Disadvantages**
1) Reduced risk of skin and knot complications 2) Ability to repair each part of the meniscus anatomically 3) Cost‐effective 4) Can be used to repair meniscal body tears	1) Technically demanding: Require some experience in SutureLasso handling and knot tying 2) The need to use an accessory portal and extra instrumentation (SutureLasso device and arthroscopic retrieval) 3) Soft‐tissue bridge: Make sure that the wires are moving freely 4) Risk of chondral injury

## DISCLOSURES

The authors (Z.E.Q., W.A‐A., M.M., B.E.) declare that they have no known competing financial interests or personal relationships that could have appeared to influence the work reported in this article.

## References

[1] Twomey‐Kozak J , Jayasuriya CT . Meniscus repair and regeneration: A systematic review from a basic and translational science perspective. Clin Sports Med. 2020;39:125‐163. 10.1016/j.csm.2019.08.003 PMC6884076 31767102

[2] Kang DG , Park YJ , Yu JH , Oh JB , Lee DY . A systematic review and meta‐analysis of arthroscopic meniscus repair in young patients: Comparison of all‐inside and inside‐out suture techniques. Knee Surg Relat Res. 2019;31:1‐11. 10.5792/ksrr.17.078 PMC6425899 30497231

[3] Golz AG , Mandelbaum B , Pace JL . All‐inside meniscus repair. Curr Rev Musculoskelet Med. 2022;15:252‐258. 10.1007/s12178-022-09766-3 PMC9276856 35661090

[4] Chand SB , Santhosh G , Saseendran A , et al. Efficacy and long‐term outcomes of arthroscopic meniscus repair: A systematic review and meta‐analysis. Cureus. 2024;16:e70828. 10.7759/cureus.70828 PMC11531707 39493053

[5] Sihvonen R , Paavola M , Malmivaara A , et al. Arthroscopic partial meniscectomy for a degenerative meniscus tear: A 5 year follow‐up of the placebo‐surgery controlled FIDELITY trial. Br J Sports Med. 2020;54:1332‐1339. 10.1136/bjsports-2020-102813 PMC7606577 32855201

[6] Xu C , Zhao J. A meta‐analysis comparing meniscal repair with meniscectomy in the treatment of meniscal tears: The more meniscus, the better outcome? Knee Surg Sports Traumatol Arthrosc. 2015;23:164‐170. 10.1007/s00167-013-2528-6 23670128

[7] Milliron EM , Magnussen RA , Cavendish PA , Quinn JP , DiBartola AC , Flanigan DC . Repair of radial meniscus tears results in improved patient‐reported outcome scores: A systematic review. Arthrosc Sports Med Rehabil. 2021;3:e967‐e980. 10.1016/j.asmr.2021.03.002 PMC8220631 34195666

[8] Yuen WLP , Kuo CL , Goh KMJ , Lee YHD. Technique for all‐inside repair of bucket‐handle meniscus tears. Arthrosc Tech. 2021;10:e743‐e750. 10.1016/j.eats.2020.10.063 PMC7953237 33738210

[9] Joshi A , Basukala B , Bista R , Sharma R , Singh N , Pradhan I . Medial meniscus repair using “AJStitch”: A cost‐effective all‐inside all‐suture meniscal repair system. Arthrosc Tech. 2024;13:103106. 10.1016/j.eats.2024.103106 PMC11662861 39711901

